# Mechanisms of Gain Control by Voltage-Gated Channels in Intrinsically-Firing Neurons

**DOI:** 10.1371/journal.pone.0115431

**Published:** 2015-03-27

**Authors:** Ameera X. Patel, Denis Burdakov

**Affiliations:** 1 Brain Mapping Unit, University of Cambridge, Cambridge, UK; 2 MRC National Institute for Medical Research, London, UK; 3 MRC Centre for Developmental Neurobiology, King’s College London, London, UK; University Paris 6, FRANCE

## Abstract

Gain modulation is a key feature of neural information processing, but underlying mechanisms remain unclear. In single neurons, gain can be measured as the slope of the current-frequency (input-output) relationship over any given range of inputs. While much work has focused on the control of basal firing rates and spike rate adaptation, gain control has been relatively unstudied. Of the limited studies on gain control, some have examined the roles of synaptic noise and passive somatic currents, but the roles of voltage-gated channels present ubiquitously in neurons have been less explored. Here, we systematically examined the relationship between gain and voltage-gated ion channels in a conductance-based, tonically-active, model neuron. Changes in expression (conductance density) of voltage-gated channels increased (Ca^2+^ channel), reduced (K^+^ channels), or produced little effect (h-type channel) on gain. We found that the gain-controlling ability of channels increased exponentially with the steepness of their activation within the dynamic voltage window (voltage range associated with firing). For depolarization-activated channels, this produced a greater channel current per action potential at higher firing rates. This allowed these channels to modulate gain by contributing to firing preferentially at states of higher excitation. A finer analysis of the current-voltage relationship during tonic firing identified narrow voltage windows at which the gain-modulating channels exerted their effects. As a proof of concept, we show that h-type channels can be tuned to modulate gain by changing the steepness of their activation within the dynamic voltage window. These results show how the impact of an ion channel on gain can be predicted from the relationship between channel kinetics and the membrane potential during firing. This is potentially relevant to understanding input-output scaling in a wide class of neurons found throughout the brain and other nervous systems.

## Introduction

Gain control is a central unsolved problem in the biophysics of neural computation. The ability of neurons to modulate gain is a fundamental feature of neural information processing [1, 2], yet our understanding of the underlying biophysical mechanisms is currently limited. While much work has centered around factors that can change the basal firing rate of neurons, and on spike rate adaptation, including the classical work of Connor and Stevens on the A-current [3], gain control has been relatively unstudied.

Gain represents the degree of scaling between the input and output of a system, often quantified as the slope of the relationship between the input and output over any given range of input magnitudes. In single neurons, the input can be measured in a number of ways including, but not limited to, the magnitude of stimulatory current or excitatory conductance, the excitatory pre-synaptic firing rate, or the strength of a functional stimulus. The resulting output of the neuron can then be quantified, simply, by the neuronal firing rate or spiking probability. Factors that can effect a shift from a neuron’s tuning curve (the baseline input-output relationship) to another, thus modulating the gain, may critically underlie many neurophysiological and pathological neural processes. Understanding these factors is thus critical for understanding brain function [1, 4].

The electrical activity of a neuron is a product of the interaction between membrane currents from synaptic stimuli, currents from ion channels intrinsically active in the membrane [5], intrinsic passive properties of the neuron, and dendritic structure. While a number of studies have shown that the input-output relationship of a neuron can be critically affected by synaptic tone [4, 6–8], demonstrations of the roles of intrinsic ionic conductances in gain modulation have been limited. Voltage-gated currents, as well as background ‘passive’ currents, are of particular interest because they are present ubiquitously in most neurons [9]. Although a limited number of theoretical and experimental studies suggest that some of these channels may critically affect the gain of a neuron [5, 10–12], a systematic analysis of the effects of individual ion channels on gain has not yet been performed. Furthermore, there have been no studies that detail an underlying general mechanism by which changes in ionic conductances can tune the gain of a neuron.

Here, we systematically explored the relationship between gain and the activity of several archetypal voltage-gated and passive ion channels in a Hodgkin-Huxley-type model neuron. Through analyzing the effects of current flowing through these channels on the action potential and inter-spike interval, which is controlled by intrinsic properties of the channels, we propose a new mechanism by which these voltage-gated channels can control the gain of the neuron by influencing the computation of the inter-spike interval. These results are important in understanding how changes in channel densities, or indeed channel activation properties, in physiological or pathological situations, can translate to changes in the way neurons respond to inputs and thus affect neural network activity.

## Materials and Methods

### Model neuron

We used a Hodgkin-Huxley-type single compartment model neuron, based on lobster somatogastric neurons [13, 14], as in our previous studies [10, 15]. This model comprises seven voltage-gated membrane channels (a fast sodium channel, *I*_*Na*_; a fast, *I*_*CaT*_, and slow, *I*_*CaS*_, calcium channel; a fast and transient potassium channel, also known as the A-type channel, *I*_*A*_; a calcium-activated potassium channel, *I*_*KCa*_; a delayed-rectifier potassium channel, *I*_*Kd*_; and a hyperpolarization-activated inward channel, also known as the h-type channel, *I*_*h*_), a voltage-independent leak channel, *I*_*leak*_, and an intracellular calcium buffer [14].

Each channel’s current, *I*_*ion*_, was defined in the model as a current density (in *μ*A⋅cm^−2^) according to the following general equation, as described in the Hodgkin-Huxley model:

$$\begin{array}{c} I_{ion}={\bar{G}}_{ion}m^{\rho }h\left(V-E_{ion}\right), \end{array}$$ (1)

where ${\bar{G}}_{ion}$ represents the maximal specific conductance of the channel in mS⋅cm^−2^, *V* represents the membrane potential in mV, *E*_*ion*_ is the reversal potential in mV, *m* and *h* are the activation and inactivation variables respectively, and *ρ* represents the postulated number of gates.

The activation, *m*, and inactivation, *h*, variables for each channel were defined by the following set of differential equations:

$$\begin{array}{c} \frac{dm}{dt}=\frac{m_{\infty }-m}{\tau_{m}}\;\text{and}\;\frac{dh}{dt}=\frac{h_{\infty }-h}{\tau_{h}}. \end{array}$$ (2)

The equations defining the activation and inactivation time constants (*τ*_*m*_ and *τ*_*h*_), and the steady-state values of the activation and inactivation variables (*m*_∞_ and *h*_∞_) for each channel can be found in Table 1. For the non-voltage-gated leak channel (*I*_*leak*_), current density was defined by Ohm’s Law, which is equivalent to Equation 1 without the activation and inactivation terms:

$$\begin{array}{c} I_{ion}={\bar{G}}_{ion}\left(V_{m}-E_{ion}\right). \end{array}$$ (3)

**Table 1:** Activation and inactivation kinetics of voltage-gated channels. *ρ* represents the postulated number of gates, *m*_∞_ is the steady-state activation variable, *τ*_*m*_ is the activation time constant, *h*_∞_ is the steady-state inactivation variable, and *τ*_*h*_ is the inactivation time constant. The seven voltage-gated ion channels are: a fast sodium channel (*I*_*Na*_), a fast (*I*_*CaT*_) and slow (*I*_*CaS*_) calcium channel, a fast and transient potassium channel (A-type channel, *I*_*A*_), a calcium-activated potassium channel (*I*_*KCa*_), a delayed-rectifier potassium channel (*I*_*Kd*_), and a hyperpolarization-activated inward channel (h-type channel, *I*_*h*_).

	*ρ*	*m*_∞_	*τ*_*m*_	*h*_∞_	*τ*_*h*_
*I*_*Na*_	3	$\frac{1}{1+e^{\left(\frac{V+25.5}{-5.9}\right)}}$	$2.64-\frac{2.52}{1+e^{\left(\frac{V+120}{-25}\right)}}$	$\frac{1}{1+e^{\left(\frac{V+48.9}{5.18}\right)}}$	$\frac{1.34}{1+e^{\left(\frac{V+62.9}{-10}\right)}}\cdot \left(1.5+\frac{1}{1+e^{\left(\frac{V+34.9}{-3.6}\right)}}\right)$
*I*_*CaS*_	3	$\frac{1}{1+e^{\left(\frac{V+33}{-8.1}\right)}}$	$2.8+\frac{14}{e^{\left(\frac{V+27}{10}\right)}+e^{\left(\frac{V+70}{-13}\right)}}$	$\frac{1}{1+e^{\left(\frac{V+60}{6.2}\right)}}$	$120+\frac{300}{e^{\left(\frac{V+55}{9}\right)}+e^{\left(\frac{V+65}{-16}\right)}}$
*I*_*CaT*_	3	$\frac{1}{1+e^{\left(\frac{V+27.1}{-7.2}\right)}}$	$43.4-\frac{42.6}{1+e^{\left(\frac{V+68.1}{-20.5}\right)}}$	$\frac{1}{1+e^{\left(\frac{V+32.1}{5.5}\right)}}$	$210-\frac{179.6}{1+e^{\left(\frac{V+55}{-16.9}\right)}}$
*I*_*A*_	3	$\frac{1}{1+e^{\left(\frac{V+27.2}{-8.7}\right)}}$	$23.2-\frac{20.8}{1+e^{\left(\frac{V+32.9}{-15.2}\right)}}$	$\frac{1}{1+e^{\left(\frac{V+56.9}{4.9}\right)}}$	$77.2-\frac{58.4}{1+e^{\left(\frac{V+38.9}{-26.5}\right)}}$
*I*_*KCa*_	4	$\frac{\left[Ca\right]}{[Ca]+3}\cdot \frac{1}{1+e^{\left(\frac{V+28.3}{-12.6}\right)}}$	$180.6-\frac{150.2}{1+e^{\left(\frac{V+46}{-22.7}\right)}}$		
*I*_*Kd*_	4	$\frac{1}{1+e^{\left(\frac{V+12.3}{-11.8}\right)}}$	$14.4-\frac{12.8}{1+e^{\left(\frac{V+28.3}{-19.2}\right)}}$		
*I*_*h*_	1	$\frac{1}{1+e^{\left(\frac{V+75}{5.5}\right)}}$	$\frac{2}{e^{\left(\frac{V+169.7}{-11.6}\right)}+e^{\left(\frac{V+26.7}{14.3}\right)}}$		

In addition to the above channels, for the simulations in Fig. 4 we introduced passive inhibitory, *I*_*i*_ (*E*_*i*_ = -90 mV), or excitatory, *I*_*e*_ (*E*_*e*_ = -20 mV), channels. As for the leak channel, these non-voltage-gated currents were defined by Equation 3. A summary of the baseline maximal specific conductances for all channels (${\bar{G}}_{ion}$), along with their reversal potentials (*E*_*ion*_) can be found in Table 2. These conductances are equivalent to the baseline conductances used in [14] for the tonically-firing model neuron. The reversal potential for calcium currents (*E*_*CaS*_ and *E*_*CaT*_) varied dynamically with the intracellular calcium concentration ([Ca]_*i*_), as determined by the Nernst equation at a temperature *T* of 310 K (36.85*°*C), and an extracellular calcium concentration ([Ca]_*o*_) of 3 mM (see Table 2).

**Table 2:** Baseline properties of membrane currents. *R* is the universal gas constant (8.3145 J· K^1^·mol^1^), *T* is the temperature (310 K, equivalent to 36.85*°*C), *z* is the number of moles transferred (1 mol), and *F* is the Faraday constant (96485 C·mol^1^). The extracellular calcium concentration [Ca^2+^]_*o*_ was fixed at 3 mM.

	${\bar{G}}_{ion}$ (mS·cm^−2^)	*E*_*ion*_ (mV)
*I*_*Na*_	200	+50
*I*_*CaS*_	4	$\frac{RT}{zF}\cdot log\frac{{\left[Ca\right]}_{o}}{{\left[Ca\right]}_{i}}$
*I*_*CaT*_	0	$\frac{RT}{zF}\cdot log\frac{{\left[Ca\right]}_{o}}{{\left[Ca\right]}_{i}}$
*I*_*A*_	10	–80
*I*_*KCa*_	10	–80
*I*_*Kd*_	125	–80
*I*_*h*_	0.05	–20
*I*_*leak*_	0.04	–50
*I*_*e*_	0	–20
*I*_*i*_	0	–90

### Depolarizing stimuli

**Fig 1:**
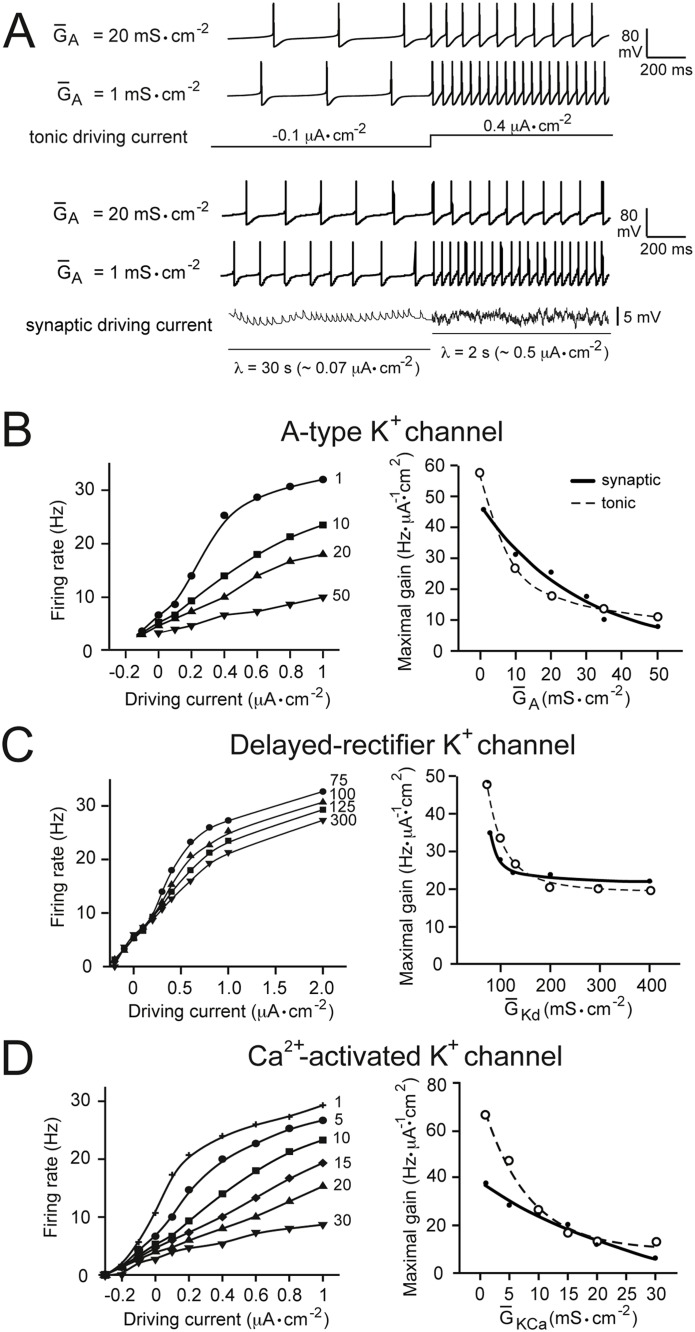
The effects of modulating voltage- and Ca^2+^-gated K^+^ conductances on gain. (A) Examples of firing responses of the model neuron with different values of A-type channel maximal specific conductance (${\bar{G}}_{A}$). The input stimuli driving the firing rate are shown schematically below the traces: current for the tonic input model (upper panel) and membrane potential for the synaptic input model (lower panel). Positive values of tonic driving current represent depolarizing input. *λ* is the mean interval between impulses in the synaptic input model (see Methods). (B) The left panel shows current-frequency (input-output) relationships obtained with different values of ${\bar{G}}_{A}$ in the model neuron (maximal specific conductance densities, in mS·cm^−2^, are given near the corresponding tuning curves) when stimulated with different tonic driving current magnitudes. The right panel shows data in the left panel re-plotted as maximal gain (see Methods) against ${\bar{G}}_{A}$ for the tonic driving input (dotted line) and the fluctuating synaptic input (solid line). (C) The same analysis shown in panel B, for the delayed-rectifier K^+^ channel, and (D) the Ca^2+^-activated K^+^ channel. Increasing maximal specific conductances of all three K^+^ channels reduced neuronal gain in response to both tonic and fluctuating inputs.

**Fig 2:**
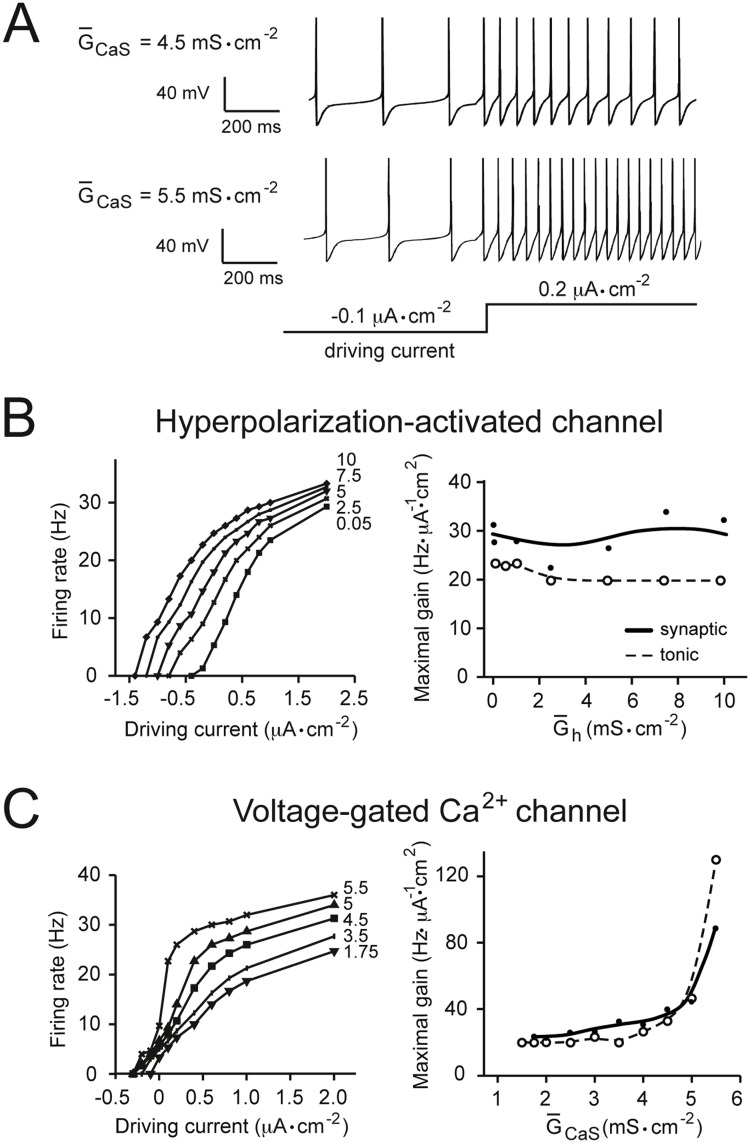
The effects of modulating voltage-gated Ca^2+^ and hyperpolarization-activated conductances on gain. (A) Examples of firing responses of the model neuron with different values of voltage-gated Ca^2+^ channel maximal conductance (${\bar{G}}_{CaS}$). The input driving current (here a tonic stimulus) is shown schematically below the traces. Positive values of driving current represent depolarizing input. (B) The left panel shows current-frequency (input-output) relationships obtained with different values of the maximal specific conductance, ${\bar{G}}_{h}$, of the hyperpolarization-activated inward channel (h-type channel), when stimulated with an increasing tonic driving current. Maximal specific conductance densities, in mS·cm^−2^, are given near the corresponding tuning curves. The right panel shows data in the left panel re-plotted as maximal gain (see Methods) against ${\bar{G}}_{h}$ for the tonic driving input (dotted line) and for the fluctuating synaptic input (solid line). (C) The same analysis shown in panel B for the voltage-gated Ca^2+^ channel. Increasing ${\bar{G}}_{h}$ appeared to have no effect on gain, but increasing ${\bar{G}}_{CaS}$ increased gain in response to both tonic and fluctuating inputs.

For simulations where we introduced an external input stimulus, this was in one of two forms: (i) a tonic, sustained, driving current of variable intensity, or (ii) a fluctuating, current-based, synaptic input [15]. For analyses in Figs. 1, 2, 4 and 5, the tonic stimulus was incrementally increased up to a maximum of 2 *μ*A·cm^−2^. The synaptic model introduced a series of 2 ms impulses of magnitude 0.75 *μ*A·cm^−2^, separated by Poisson-distributed random intervals (mean=*λ*). The intensity of synaptic input was varied by modulating the mean inter-pulse interval, *λ*, between 30 ms and 0.5 ms [15].

### Membrane potential dynamics

The membrane potential was governed by all membrane currents (both voltage-gated and non-voltage-gated) according to the following equation:

$$\begin{array}{c} C_{m}\cdot \frac{dV}{dt}=\sum_{i\in I_{T}}I_{i}, \end{array}$$ (4)

where *I*_*T*_ represents the set of membrane current densities, *C*_*m*_ represents the specific capacitance of the membrane, and *dV*/*dt* represents the fluctuation of membrane potential with time. *C*_*m*_ is generally assumed to lie within the range of 0.4 − 1 *μ*F·cm^−2^ [5, 16–18], and was fixed at 0.6 *μ*F·cm^−2^ in our model. We found no effect of varying membrane capacitance within this range on gain control, and include further analysis of this in Supplementary S1 Fig. The membrane potential dynamics were computed using MATLAB stiff systems numerical integrator ode23s, with a time resolution of 250 *μ*s.

Analysis in this paper was restricted to tonically-firing cells, which correspond, biologically, to a large group of cells including (among others) midbrain dopaminergic neurons [19], thalamic neurons [20], serotonergic neurons of the raphe [21], and several classes of widely-projecting hypothalamic neurons [22] and hypothalamic interneurons [23]. In analyses where conductances were varied from their baseline values (in Table 2), these changes were constrained to keep the neuronal firing pattern tonic and regular, and to avoid silence or bursts.

### Calcium dynamics

The intracellular calcium concentration ([Ca^2+^]_*i*_) that controlled *E*_*CaS*_, *E*_*CaT*_ and *I*_*KCa*_, was defined by a differential equation similar to the Hodgkin-Huxley equations characterizing activation and inactivation variables for voltage-gated channels. This equation models the processes of calcium diffusion, buffering and sequestration as a change in effective intracellular calcium concentration through time, using an exponentially decaying process [24]. In our simulations, the effective intracellular calcium concentration was determined by integrating the following equation [14]:

$$\begin{array}{c} \frac{d{\left[Ca^{2+}\right]}_{i}}{dt}=\frac{-f\left(I_{CaT}+I_{CaS}\right)-{\left[Ca^{2+}\right]}_{i}+{\left[Ca^{2+}\right]}_{b}}{\tau_{Ca^{2+}}}, \end{array}$$ (5)

where *τ*_*Ca*^2+^_ is the calcium pool removal time constant, set at 200 ms, *f* is the factor that translates current density into a concentration, set at 14.96 M·A^−1^·cm^2^, and [Ca^2+^]_*b*_ is the baseline intracellular calcium concentration, set at 0.05 *μ*M.

For simulations where I_*KCa*_ was uncoupled from changes in intracellular calcium concentration, the calcium term in the channel’s steady-state activation variable (m_∞_) was fixed at a value of 0.015 mM (see Table 1). This corresponded to the mean intracellular calcium concentration at the baseline firing rate of 5 Hz (channel conductances as in Table 2), which is within a physiological range of [Ca^2+^] measured in microdomains around voltage-gated Ca^2+^ channels [25]. In Fig. 5C,D, the strength of coupling between *I*_*CaS*_ and *I*_*KCa*_ was controlled by multiplying the activation variable equation for *I*_*KCa*_ (Table 1, m_∞_) by a numerical coupling coefficient, referred to as the ‘strength of coupling’. The values of this coefficient are stated in the relevant panels of Fig. 5.

### Gain calculation

Gain represents the slope of the driving current (input) *vs.* firing frequency (output) relationship at any given point along the curve. Given that gain varies as a function of the driving current or firing frequency, a summary measure of gain was required for each tuning curve (input-output relationship), such as the mean or maximal gain. Since the former relies heavily on the current range over which the simulations are conducted, for example, simulating over a wide range of input currents will make the measure less sensitive to large changes in gain over a specific narrow current range, we used the maximal gain. To compute this, for each set of input-output values a spline model was fitted to each curve (except for synaptic input data where a third order polynomial function was used due to between-simulation variability in output), and the maximal derivative was calculated. We refer to this value in the Results and Figures as ‘gain’, measured in Hz·*μ*A^−1^·cm^2^.

### Average current per action potential

In analyses where the average current per action potential was calculated (Fig. 7) for current flowing through particular channels, current values were averaged over 10 action potentials (including the corresponding inter-spike intervals) at a time resolution of 250 *μ*s.

## Results

### Modulating the maximal specific conductance of voltage-gated channels can effect substantial gain control

To explore the effect of intrinsic conductances on gain, we first examined the firing responses of the conductance-based model neuron to increasing driving input, while changing the maximal specific conductance of each channel (${\bar{G}}_{ion}$, see Methods). In the first set of simulations, the driving current was tonic (positive values in the figures represent a depolarizing current), which mimics the most common experimental way of measuring current-frequency relationships [4, 11]. In the second set of simulations, we tested the robustness of these results with a more physiological driving current: a fluctuating, depolarizing, ‘synaptic’ input (see Methods, [15]).

We found that increases in the ${\bar{G}}_{ion}$ of all voltage-gated K^+^ channels—the A-type channel (*I*_*A*_), the delayed-rectifier K^+^ channel (*I*_*Kd*_), and the Ca^2+^-activated channel (*I*_*KCa*_)—caused a reduction in gain (Fig. 1). This effect was observed with both the tonic driving input (Fig. 1B-D, right side panels, dotted lines) and the synaptic input (Fig. 1B-D, right side panels, solid lines), though was more marked for the tonic driving stimulus.

In contrast, we found more diverse effects on gain from changes in ${\bar{G}}_{ion}$ of the slow voltage-gated Ca^2+^ channel (*I*_*CaS*_) and the hyperpolarization-activated inward channel, or h-type channel (*I*_*h*_). Increasing ${\bar{G}}_{h}$, even up to 20 times its baseline value produced no change in the maximal gain of the input-output relationship (Fig. 2B). This effect was observed for the tonic driving stimulus (Fig. 2B, right panel, dotted line), and the synaptic input model (Fig. 2B, right panel, solid line). Increasing ${\bar{G}}_{CaS}$, on the other hand, caused a potent increase in maximal gain. This gain-increasing effect was greatest above a ${\bar{G}}_{CaS}$ of 4.5 mS·cm^−2^, and was again observed in response to both tonic current and synaptic inputs (Fig. 2C).

**Fig 3:**
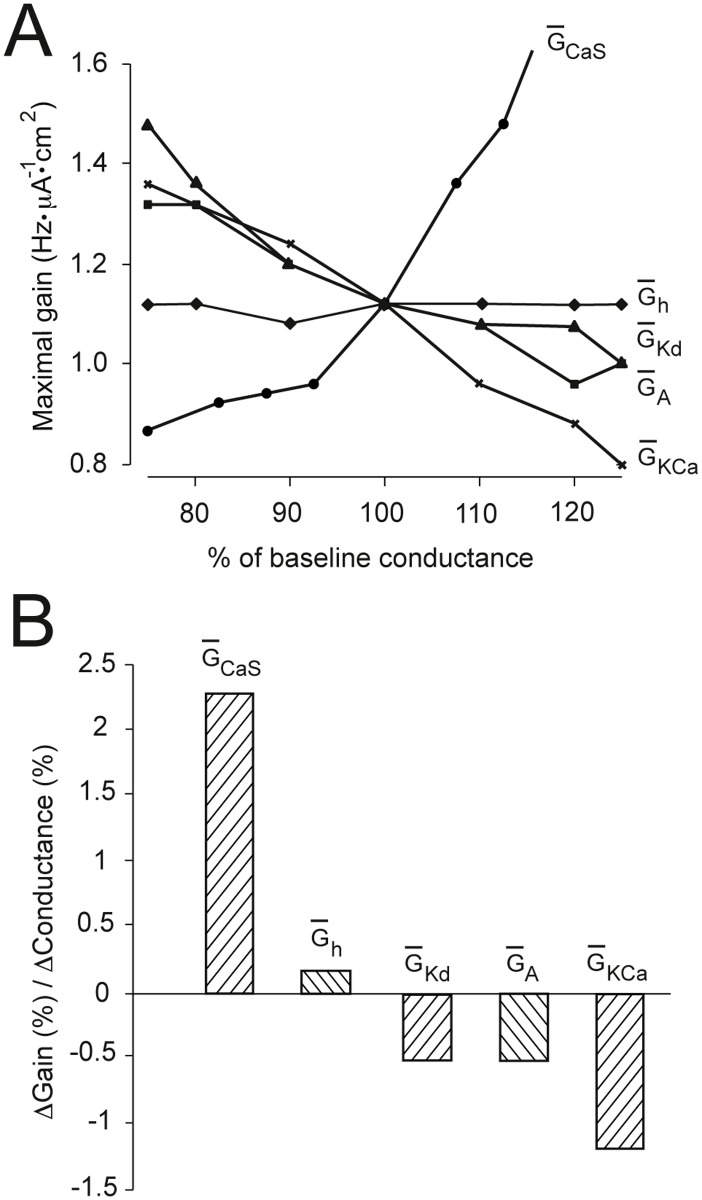
The effects of small changes in channel conductance on neuronal gain. (A) Maximal gain (see Methods) calculated as in Figs. 1 and 2 plotted against the percentage change from the baseline maximal specific conductance (see Table 2) of each channel. (B) Gradients of the lines shown in panel A, at the 100% conductance point, showing the relative impact of changes in maximal conductance on gain for each voltage-gated channel.

Next, we analyzed the gain-modulating ability of these channels from small changes in their ${\bar{G}}_{ion}$ value. Under physiological conditions in real neural circuits, the ${\bar{G}}_{ion}$ values of voltage-gated channels are not likely to vary through as large a range as in the above simulations. Experiments suggest that upon physiological modulation in real neurons, voltage-gated conductances are not likely to deviate from their resting value by more than ∼ 30% [26–28]. To compare the impact of small changes in voltage-gated ${\bar{G}}_{ion}$ on maximal gain, we computed maximal gain as a function of the percent change in maximal conductance (Fig. 3A, see Table 2 for baseline ${\bar{G}}_{ion}$ values). Consistent with our previous simulations, these results revealed that the *I*_*CaS*_ channel was the most potent gain modulator. In contrast, increasing ${\bar{G}}_{h}$ had a negligible gain-increasing effect, and increasing the maximal conductances of the voltage-gated K^+^ channels had moderate gain-reducing effects, with *I*_*KCa*_ being the most potent gain-reducer of the three K^+^ channels (Fig. 3).

### The maximal specific conductance of passive channels has no effect on gain

**Fig 4:**
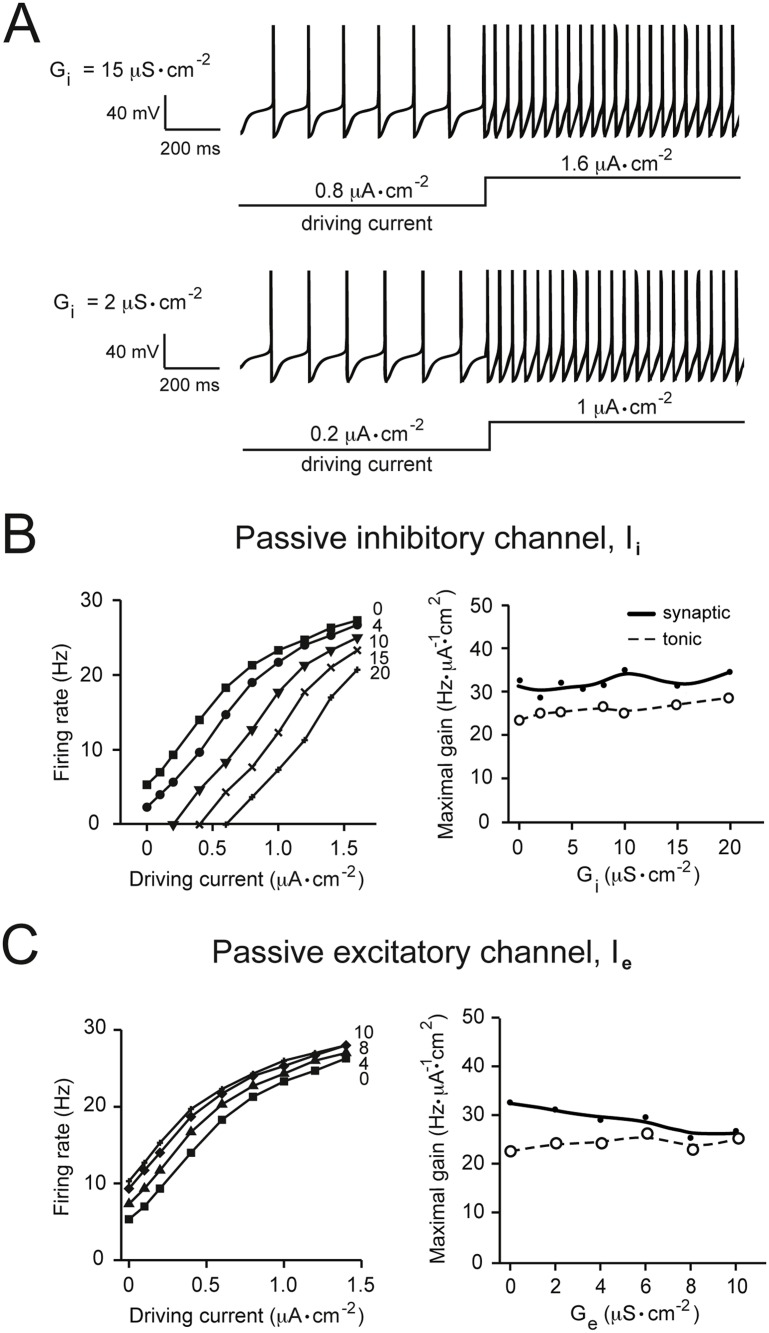
The effects of passive membrane conductances on gain. (A) Examples of firing responses of the model neuron with different maximal specific conductance densities of the passive (non-voltage-gated) inhibitory channel (${\bar{G}}_{i}$). The current step (tonic depolarizing current input) is of the same amplitude in the two traces, but the baselines were shifted to points giving the same firing rates for visual clarity. (B) The left panel shows current-frequency (input-output) relationships obtained with different values of ${\bar{G}}_{i}$ when stimulated with an increasing tonic driving current. Inward, depolarizing, driving currents are represented as positive values. Maximal specific conductance densities, in *μ*S·cm^−2^, are given near the corresponding tuning curves. The right panel shows data in the left panel re-plotted as maximal gain (see Methods) against ${\bar{G}}_{i}$ for the tonic driving input (dotted line) and for the fluctuating synaptic input (solid line). (C) The same analysis shown in panel B for the passive excitatory channel. Increasing ${\bar{G}}_{e}$ or ${\bar{G}}_{i}$ appeared to have no effect on gain in response to both tonic and fluctuating inputs.

Passive channels which act as ohmic conductors have generally been assumed to cause parallel shifts in the input-output relationship. In other words, increasing their ${\bar{G}}_{ion}$ would not be expected to change the slope of the neuronal tuning curve, and would therefore not be expected to modulate gain [4, 6, 29–31]. However, the generality of this assumption has recently been questioned by experimental demonstrations that an artificially injected current can modulate gain in some neurons [11, 32]. We have therefore re-examined this issue in our model neuron. For this we introduced a passive inhibitory channel (*I*_*i*_) into the model and, as in the above simulations, drove an increase in the firing rate with both a tonic driving stimulus and a fluctuating synaptic input. This was repeated for different ${\bar{G}}_{i}$ values, and the maximal gain for each tuning curve was calculated. As predicted, we found no effect of increasing the passive inhibitory conductance on the maximal gain of the neuron for both types of driving stimuli (Fig. 4B). We repeated these simulations for a passive excitatory channel (*I*_*e*_) and similarly found a negligible effect of changing ${\bar{G}}_{e}$ on gain (Fig. 4C).

### The effects of channel coupling on gain control

**Fig 5:**
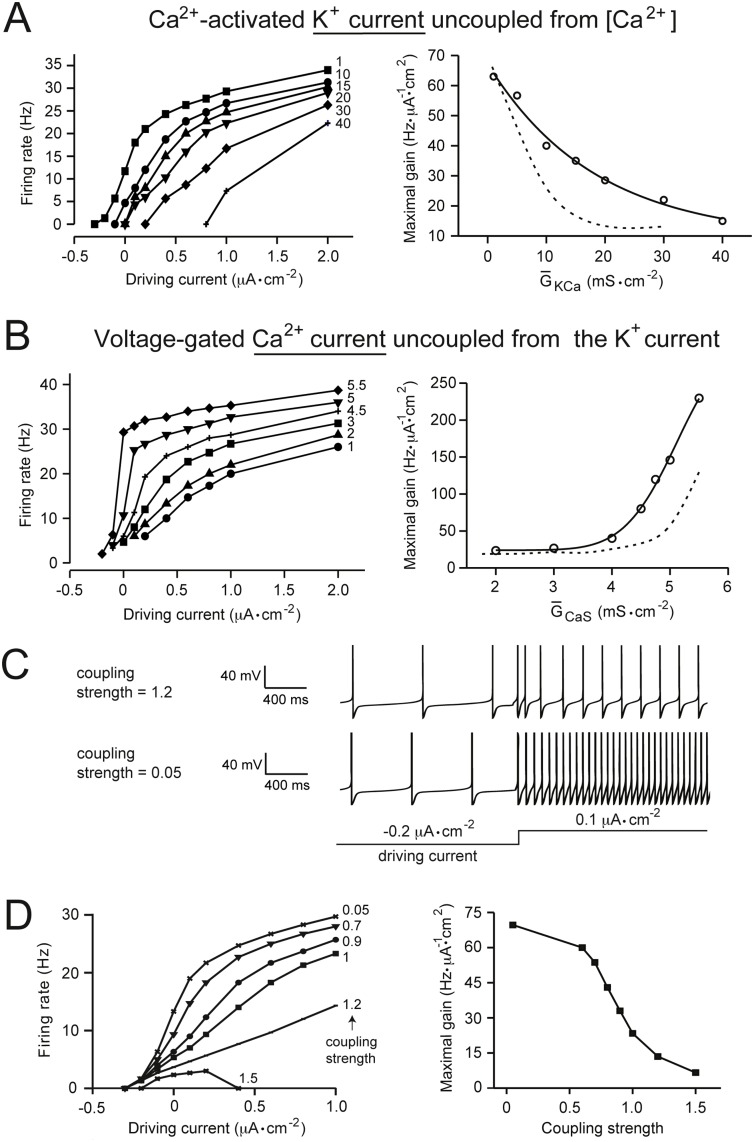
The role of Ca^2+^-dependent coupling between voltage-gated Ca^2+^ and K^+^ currents. (A) The left panel shows current-frequency (input-output) relationships obtained when varying the maximal specific conductance of the Ca^2+^-activated K^+^ channel (${\bar{G}}_{KCa}$) when uncoupled from changes in intracellular [Ca^2+^]. The input was a tonic driving current where positive values of the current represent depolarizing input. The right panel shows data from the left panel re-plotted as maximal gain (see Methods) against ${\bar{G}}_{KCa}$ in the uncoupled (solid line) and the default coupled (dashed line) state. (B) This panel shows the effects of uncoupling the *I*_*KCa*_ channel from [Ca^2+^] on the voltage-gated Ca^2+^ channel (*I*_*CaS*_). The analysis conducted was the same as in panel A, but while varying the maximal conductance of the *I*_*CaS*_ channel (${\bar{G}}_{CaS}$). (C) Firing responses of the neuron under different coupling strengths (see Methods). The input (tonic) driving current is shown schematically below the traces. (D) The left panel shows current-frequency relationships in response to a tonic driving current, where the *I*_*KCa*_ and *I*_*CaS*_ channels were coupled by different strengths (see Methods). The right panel shows data in the left panel re-plotted as maximal gain (see Methods) against the strength of coupling between the two channels.

In our model, and in physiological neurons, the activity of voltage-gated Ca^2+^ channels is often coupled to the activity of Ca^2+^-activated K^+^ channels (*I*_*KCa*_), as calcium ions entering the neuron through the former can bind to and modulate the latter. It is therefore important to consider the role of channel coupling in gain modulation. To analyze this, we uncoupled the *I*_*KCa*_ channel from changes in intracellular calcium, by fixing the [Ca^2+^] term in the channel’s steady-state activation variable (see Methods). We then analyzed the effects of changing ${\bar{G}}_{ion}$ on gain control by the *I*_*KCa*_ channel (Fig. 5A), and the indirect effects of channel uncoupling on gain control by the *I*_*CaS*_ channel (Fig. 5B). After uncoupling, increasing ${\bar{G}}_{KCa}$ caused a reduction in the maximal gain, and increasing ${\bar{G}}_{CaS}$ caused a potent increase in the maximal gain, as in the coupled model. However, comparison of gain control by these two conductances in the uncoupled *vs.* coupled states revealed that uncoupling these two channels caused an increased ability of the *I*_*CaS*_ channel to increase gain, and a reduced ability of the *I*_*KCa*_ channel to reduce gain (Fig. 5A,B; dotted lines coupled, solid lines uncoupled). This suggests that the physiological function of coupling between these two channels in gain control could be to moderate increases in gain.

To further analyze this, we varied the strength of coupling between the two channels (see Methods). Increasing the strength of coupling was found to have a steep gain reducing effect (Fig. 5D). Hence, although gain control by these two voltage-gated channels is a factor of their intrinsic properties (leading to gain control independently of each other), their strength of interaction is also likely to be a key factor in gain modulation and the control of excitability.

### The gain-modulating power of voltage-gated channels relates to the steepness of their activation curves within the dynamic voltage window

**Fig 6:**
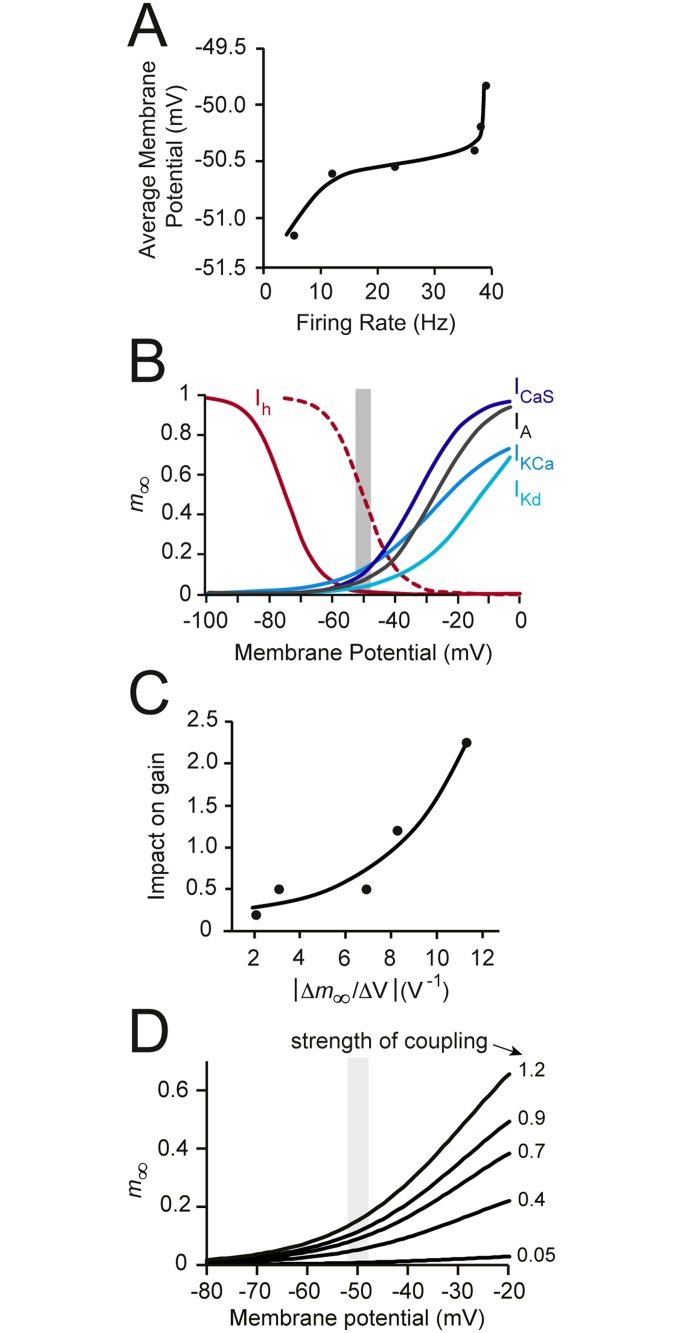
Gain modulation and the voltage dependence of channel activation. (A) The firing rate of the model neuron plotted against the average membrane potential, illustrating the ‘dynamic voltage window’ of the neuron (∼ -51 to -50 mV). (B) The voltage dependence of steady-state activation variables (*m*_∞_) used in our simulations. The shaded area shows the dynamic voltage window from panel A. The dashed line shows the shifted activation curve of *I*_*h*_ used for simulations in Fig. 10 (half-maximal activation of the shifted curve occurs at -50 mV instead of the baseline value of -75 mV). (C) The correlation between change in *m*_∞_ within the dynamic voltage window and the ‘impact on gain’ for each voltage-gated channel (% change in gain per % change in maximal specific conductance from Fig. 3B). Both x and y values are plotted as positive numbers. There is a strong positive correlation (*r* = 0.92); the fitted line shown is y = 0.134*e* ^*x*/0.004^. (D) The effects of different Ca^2+^-K^+^ channel coupling strengths (see Methods) on the steady-state activation variable (*m*_∞_) of the Ca^2+^-activated K^+^ channel. The dynamic voltage window is shaded in grey.

Given our observations that voltage-gated channels modulate gain, but non-voltage-gated channels do not, we inferred that the ability of the former to control gain must be a factor of their activation and/or inactivation kinetics. Here, we looked at channel activation. Theoretically, for a channel to effect control over gain, it must exert its effects within the ‘dynamic voltage window’ (the voltage range associated with firing [5], Fig. 6A). Put more simply, as more voltage-gated channels will be active under a greater depolarizing drive (or higher firing rate), channels that activate steeply within the dynamic voltage window will contribute more to the firing rate when the drive is larger. These channels should then preferentially act on the right side of the tuning curve, thus changing gain. Inward currents that activate strongly within this window would be expected to increase gain, and outward currents that activate strongly within this window would be expected to reduce gain. To test this hypothesis, we plotted the steady-state activation variable of each voltage-gated channel as a function of membrane potential (Fig. 6B) and measured the gradient of their activation curves within the dynamic voltage window. We then plotted these gradients (∣Δ*m*_∞_/Δ*V*∣, calculated for each channel) against the ability of that channel to impact gain through changes in ${\bar{G}}_{ion}$ (Fig. 6C). As predicted, we found that the gain-modulating ability of a channel was proportional (*r* = 0.92, Pearson correlation) to the steepness of the activation curve of that channel within the dynamic voltage window (Fig. 6C).

Next, we analyzed the position of the *I*_*KCa*_ activation variable within the dynamic voltage window under varying degrees of coupling with the *I*_*CaS*_ channel. The aim was to determine whether our findings on gain control by mutual coupling between these channels are consistent with this hypothesis. We found that strong coupling between *I*_*KCa*_ and *I*_*CaS*_ resulted in an increase in the steepness of the *I*_*KCa*_ steady-state activation variable in the dynamic voltage window (see Fig. 6D), which is consistent with the combined gain-reducing ability by increased coupling we described above (Fig. 5D). Thus, the more coupled the two channels, the greater the combined gain reducing ability, which could be explained by a greater relative contribution of the gain-reducer (*I*_*KCa*_).

### The impact of changes in the maximal conductances of voltage-gated channels on the average current per action potential

**Fig 7:**
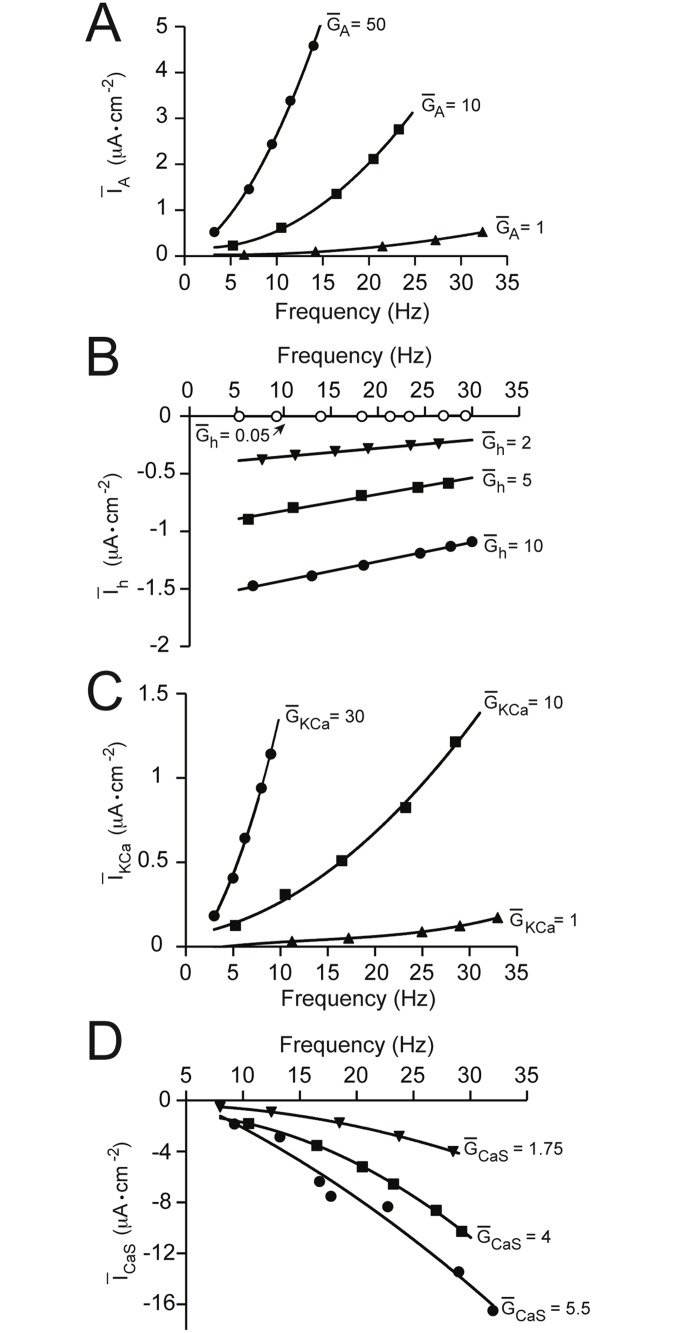
The effects of changing the maximal conductance of voltage-gated channels on the average current per action potential. This shows the average current flowing through each channel per action potential (${\bar{I}}_{ion}$, calculated over 10 action potentials, including the inter-spike intervals), plotted against firing frequency for a range of maximal specific conductance (${\bar{G}}_{ion}$) values of the: (A) A-type channel, (B) hyperpolarization-activated inward channel (h-type channel), (C) Ca^2+^-activated K^+^ channel and (D) voltage-gated Ca^2+^ channel. ${\bar{G}}_{ion}$ values adjacent to the curves are conductance densities in mS·cm^−2^. Net inward currents are represented as negative y-axis values, and outward currents as positive values.

To begin unraveling the mechanism by which changes in ${\bar{G}}_{ion}$ can modulate gain, we next analyzed channel current data, given that changes in neuronal firing rate must be transduced through changes in current. For each channel, we measured the average current per action potential (see Methods) over the range of conductances we have shown to modulate gain, and plotted this as a function of the firing frequency. Steep gradients for particular channels mean that these channels are able to conduct more current under greater depolarizing drives, or higher firing rates. As shown in Fig. 7, increasing ${\bar{G}}_{ion}$ increased the steepness of the average current per firing frequency ($\bar{I}/f$) relationship for channels which we found to modulate gain. For the outward-conducting *I*_*A*_ and *I*_*KCa*_ channels (Fig. 7A,C), this would translate to a preferential increase in outward current at higher firing rates, thus reducing gain. For the inward-conducting *I*_*CaS*_ channel (Fig. 7D), this would result in a greater inward current at higher firing rates, thus increasing gain. The parallel shifts in the $\bar{I}/f$ relationship from increases in ${\bar{G}}_{h}$ (see Fig. 7B) would, by this explanation, not result in any change in gain, which is what we observed (see Figs. 1 and 2).

### The effects of changing the maximal conductance of gain-modulating channels on the current-voltage loop

Next, we used a more fine-grain measure to look at how current flowing through these channels was changing throughout the course of an action potential, and the effects on the inter-spike interval. These new measures enabled analysis of the effects of channel activation and inactivation, as well as their time constants, in contrast to the analysis presented in section 1. Changing the shape of the inter-spike interval could result in a shift to a different tuning curve as greater (or smaller) firing rates could result from a given driving stimulus. If the effects on firing rate were more pronounced at higher firing rates (i.e. under greater depolarizing drives), this would manifest as a change in gain.

**Fig 8:**
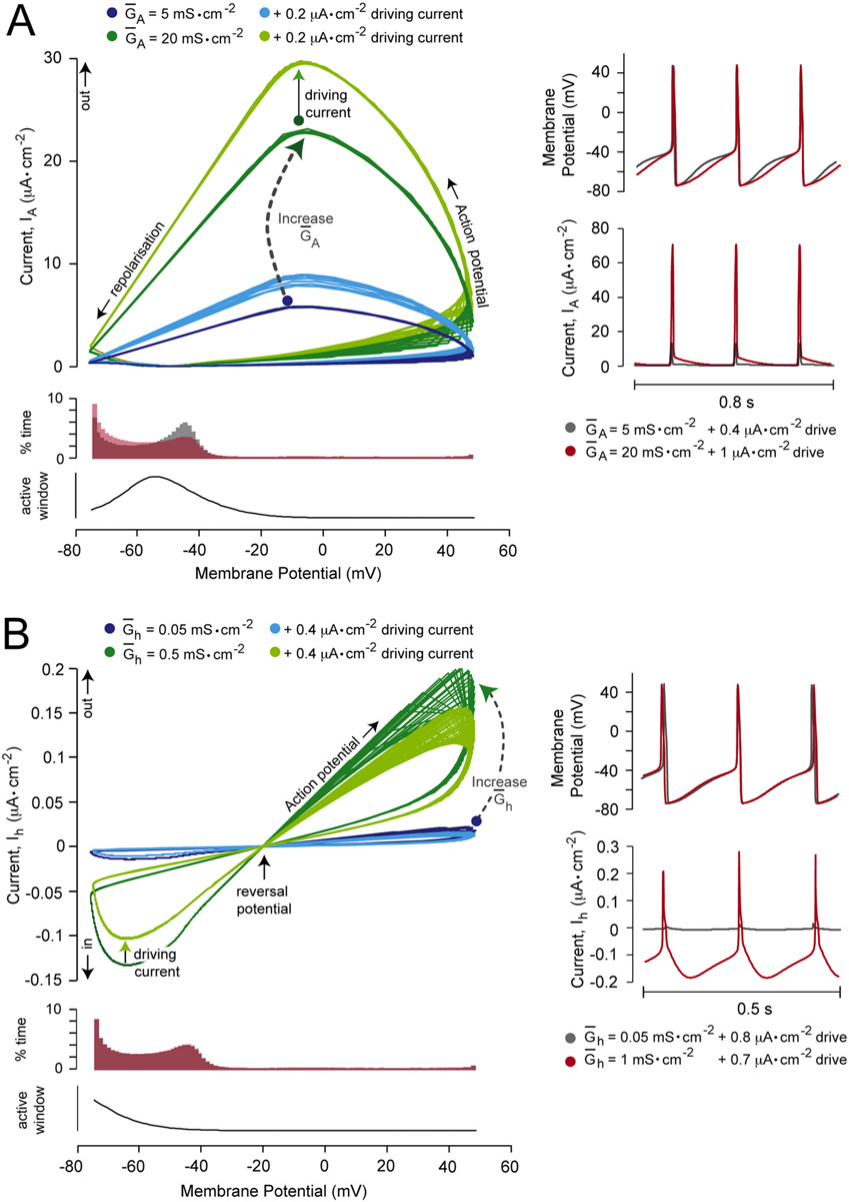
The effect of changing A- and h-type channel conductances on the current-voltage relationship. (A) The left upper panel shows a plot of A-type current against membrane potential over 4 seconds of firing (I-V loop). This relationship was plotted at different A-type channel maximal specific conductances (${\bar{G}}_{A}$) and in the presence or absence of a tonic, depolarizing, driving current. ${\bar{G}}_{A}$ and driving current values are given above the plot. The left middle panel shows histograms of the percentage time (% time) the membrane spent at various potentials over the 4 seconds of firing, for two ${\bar{G}}_{A}$ values. Firing rates were matched by changing the driving input magnitudes in order to separate the effects of changing ${\bar{G}}_{A}$ (what we were interested in), from the confounding effects of changes in firing rate as a result of changing ${\bar{G}}_{A}$. The ${\bar{G}}_{A}$ and driving current values for the histograms are given to the right of the plot. The lower left panel shows the voltages at which the channels were active (y axis: *m*_∞_ × *h*_∞_ / *τ*_*m*_ × *τ*_*h*_). The right panel shows action potential and inter-spike interval (upper) and current (lower) traces for the same ${\bar{G}}_{A}$ / driving current combinations shown in the histograms. (B) The same as panel A for the hyperpolarization-activated inward (h-type) channel, *I*_*h*_.

For the gain-reducing A-type channel (Fig. 8A), increasing either the driving stimulus or ${\bar{G}}_{A}$ caused a stretch in the A-type current *vs.* membrane potential relationship (I-V loop) along the current axis. However, increasing ${\bar{G}}_{A}$ had the additional effect of changing the percentage time spent at particular voltages. These changes were observed within the dynamic voltage window (∼-50 mV), but the % time spent at very hyperpolarized potentials (-75 to -60 mV) was also increased as the channel was also active at these voltages (Fig. 8A, lower left panel). This resulted in a change in the shape of the inter-spike interval (Fig 8A, upper right panel).

For the non-gain-modulating *I*_*h*_ channel, increasing either the driving input or ${\bar{G}}_{h}$ caused a bi-directional stretch of the I-V loop (Fig. 8B). However, increasing ${\bar{G}}_{h}$ did not change the % time spent at any voltage, as the effects of changing ${\bar{G}}_{h}$ on current were balanced inwards and outwards. Thus, there was no impact on the inter-spike interval (Fig. 8B, upper right panel).

**Fig 9:**
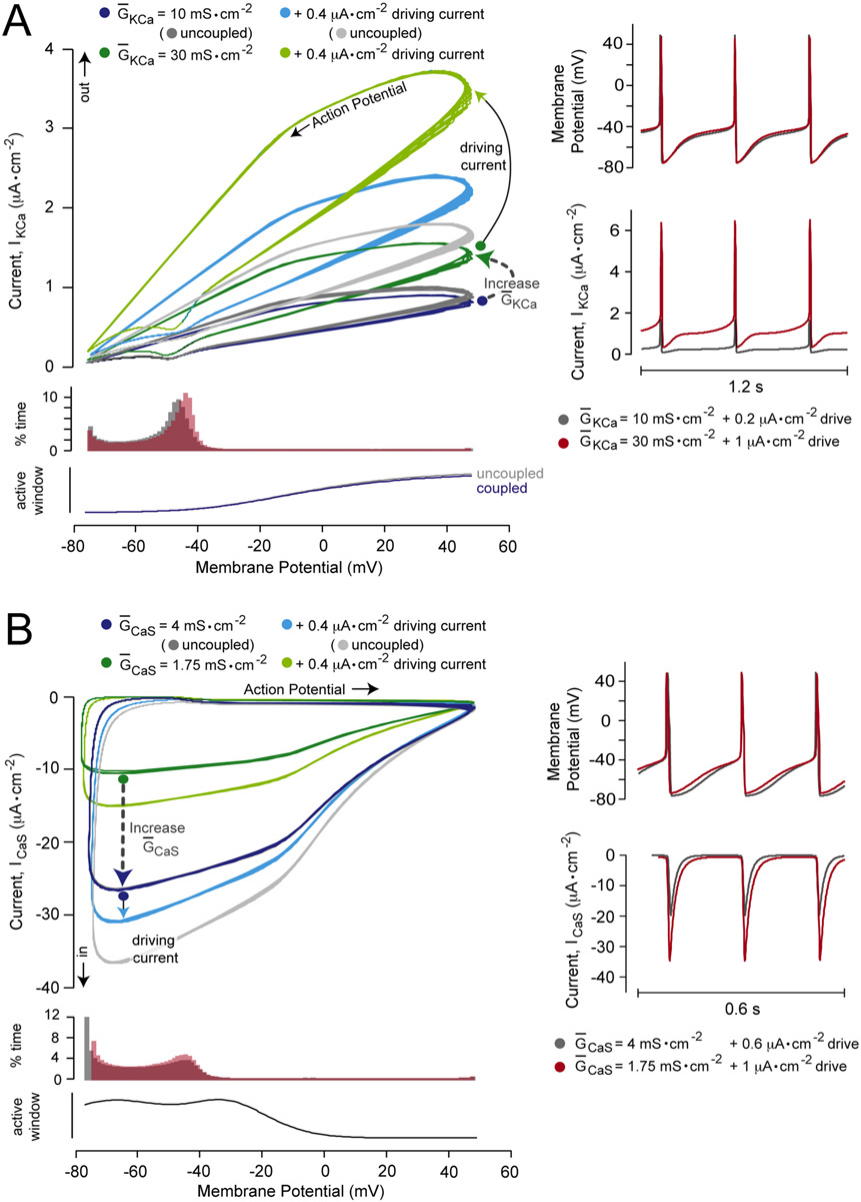
The effect of changing Ca^2+^-activated K^+^ and voltage-gated Ca^2+^ conductances on the current-voltage relationship. (A) The left upper panel shows a plot of Ca^2+^-activated K^+^ (*I*_*KCa*_) current against membrane potential over 4 seconds of firing (I-V loop). This relationship was plotted at different *I*_*KCa*_ channel maximal specific conductances (${\bar{G}}_{KCa}$) in the presence or absence of a tonic, depolarizing, driving current. The grey traces show the effect of uncoupling the *I*_*KCa*_ channel from changes in intracellular [Ca^2+^]. ${\bar{G}}_{KCa}$ and driving current values are given above the plot. The left middle panel shows histograms of the percentage time (% time) the membrane spent at various potentials over these 4 seconds. Firing rates were matched by changing the driving input magnitudes in order to separate the effects of changing ${\bar{G}}_{KCa}$ (what we were interested in), from the confounding effects of changes in firing rate as a result of changing ${\bar{G}}_{KCa}$. The ${\bar{G}}_{KCa}$ and driving current values for the histograms are given to the right of the plot. The lower left panel shows the voltages at which the channels were active (y axis: *m*_∞_ × *h*_∞_ / *τ*_*m*_ × *τ*_*h*_). The right panel shows action potential and inter-spike interval (upper) and current (lower) traces for the same ${\bar{G}}_{KCa}$ / driving current combinations shown in the histograms. (B) The same as panel A for the voltage-gated Ca^2+^ (*I*_*CaS*_) channel.

Next we looked at the gain-reducing *I*_*KCa*_ channel and the gain-increasing *I*_*CaS*_ channel, and the effects of mutual coupling between these channels. The *I*_*KCa*_ current increases preferentially at depolarized potentials. The effect of increasing the driving current or ${\bar{G}}_{KCa}$ was to cause a pivot (preferential stretch along the current axis at depolarized potentials) of the I-V loop (Fig. 9A). Increasing ${\bar{G}}_{KCa}$, however, had the additional effect of changing the % time spent around the dynamic voltage window (∼ -40 to -55 mV), represented as a notch in the I-V loop. This changed the shape of the inter-spike interval in this voltage range. Uncoupling the channel from intracellular Ca^2+^ reduced the *I*_*KCa*_ current at all membrane potentials, but most markedly in response to a depolarizing stimulus (Fig. 9A, left panel, grey traces). Based on our hypothesis, this would diminish the gain-reducing ability of the channel, which is indeed what we observed (see Fig. 5A). For the gain-increasing *I*_*CaS*_ channel, increasing either the driving current or ${\bar{G}}_{CaS}$ caused a stretch in the I-V loop along the current axis. Increasing ${\bar{G}}_{CaS}$ additionally affected the % time spent at two voltage ranges, reflecting the voltage range over which the channel was most active (Fig. 9A, lower left panel); first, around the dynamic voltage window, as we quantified in Fig. 6, and secondly at very hyperpolarized potentials (∼ -70 to -80 mV). Importantly, in the latter voltage range, increasing ${\bar{G}}_{CaS}$ enabled a prolonged inward current. The combined effect was to change the shape of the inter-spike interval between -40 and -80 mV. Uncoupling the *I*_*KCa*_ channel from changes in intracellular Ca^2+^ resulted in more *I*_*CaS*_ current in response to driving stimuli, but the I-V loop in the absence of input stimuli was unaltered from the coupled state. By our hypothesis, this would result in a greater ability to increase gain, which is what we observed (see Fig. 5B).

### Tuning a gain-neutral channel to modulate gain

**Fig 10:**
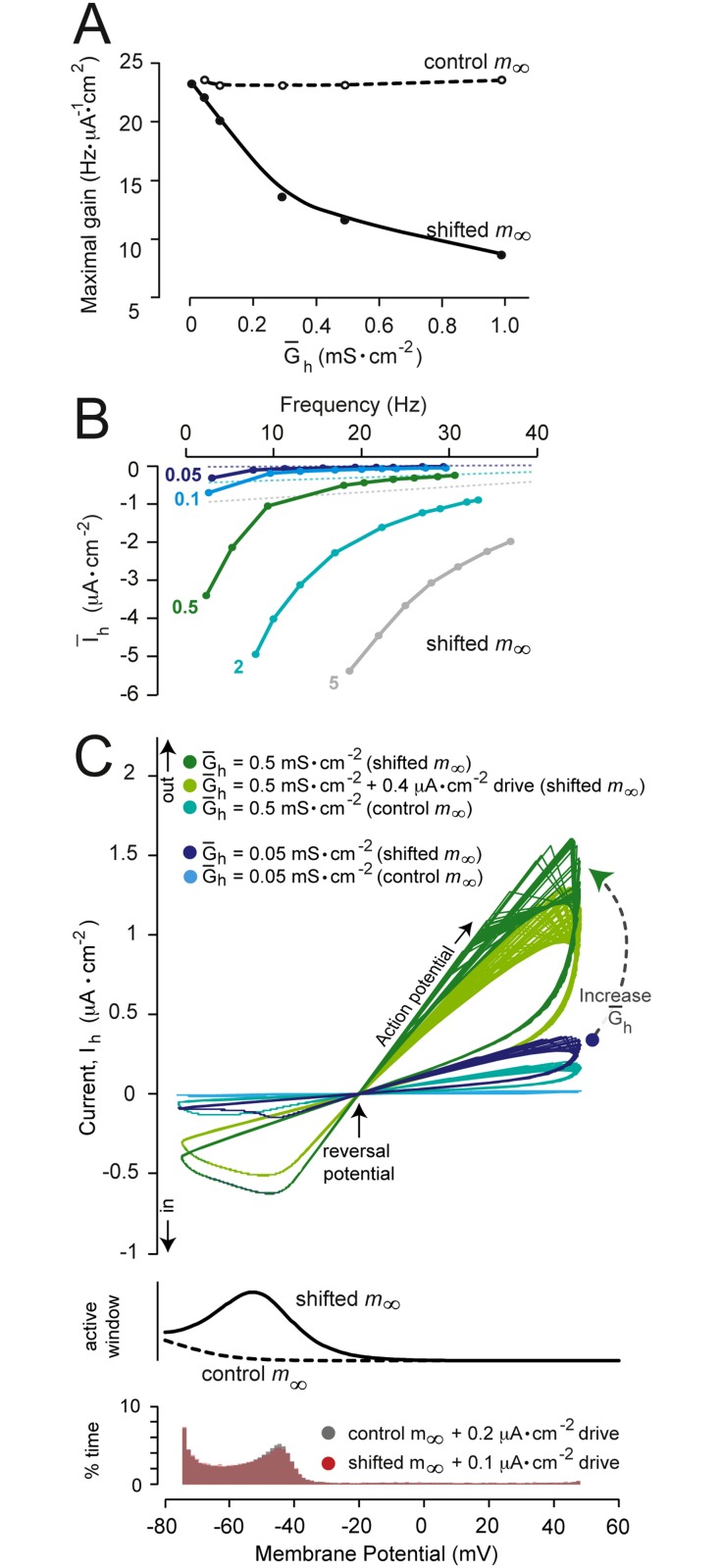
Converting a gain-neutral channel into a gain-modulating channel. (A) Shifting the steep part of the *m*_∞_ curve of *I*_*h*_ into the dynamic voltage window (as shown in Fig. 6B) enabled this, previously gain-neutral, channel to reduce gain. (B) A plot of the average *I*_*h*_ current per action potential (calculated over 10 action potentials, including the inter-spike intervals) plotted against firing frequency, for a range of maximal specific conductance (${\bar{G}}_{h}$) values of the h-type channel. Solid lines show the results for the shifted *m*_∞_ curve. Dotted lines show the results for the baseline *m*_∞_ curve (see Fig. 6B and Table 1). Maximal conductances in mS·cm^−2^ are given adjacent to the corresponding curves. (C) The upper panel shows the effect of this shift on the current-voltage relationship (I-V loop), plotted over 4 seconds of firing. ${\bar{G}}_{h}$ and driving current values are given above the plot. The left middle panel shows the voltages at which the channel was active before (control *m*_∞_) and after (shifted *m*_∞_) shifting the *m*_∞_ curve (y axis: *m*_∞_ × *h*_∞_ / *τ*_*m*_ × *τ*_*h*_). The lower panel shows histograms of the percentage time (% time) the membrane spent at various potentials over the 4 seconds of firing. Firing rates were matched by changing the driving input magnitudes to prevent differences in firing rate at different ${\bar{G}}_{h}$ magnitudes from biasing the analysis (as in Figs. 8 and 9). In summary we show that the gain-modulating properties of a channel can be controlled by modulating its activation kinetics.

As proof of concept, we conducted an experiment to validate our hypothesis. We investigated whether a non-gain-modulating channel (here the h-type channel, *I*_*h*_) could be tuned to modulate gain by shifting its activation curve along the voltage axis, such that it varied more steeply within the dynamic voltage window (Fig. 6B, dashed line, half-maximal at -50 mV). As expected, this shift afforded the *I*_*h*_ channel the ability to modulate gain (Fig. 10A). Here we observed it to reduce gain. Next we analyzed the impact of this change on the $\bar{I}/f$ relationship. As described above, we propose that the ability to modulate gain relies on an ability change the gradient of this relationship. Consistent with this, we found that increasing ${\bar{G}}_{h}$ increased the steepness of this relationship (Fig. 10B). In concert with the channel’s new gain-reducing ability, at increased ${\bar{G}}_{h}$ the channel was able to preferentially reduce its current in response to greater depolarizing drives (or higher firing rates). As a predominantly inward-conducting channel, this is what would be needed to reduce gain. Finally, we analyzed the impact of this shift in the steady-state activation curve on the *I*_*h*_ I-V loop. As demonstrated in Fig. 10C, shifting the voltage range over which the channel activated most strongly resulted in a greater depolarizing influence around the dynamic voltage window, reducing the % time the membrane spent in this voltage range. Such a change could enable the neuron to operate on a different tuning curve and allow it to reduce gain, which is what we observed (see Fig. 10A).

## Discussion

Gain control, the relationship between neuronal input and output, is a central feature of neural information processing. While the factors that affect baseline firing rates have been well characterized, stemming from the classical work of Connor and Stevens on the A-type current [3], our understanding of the factors that affect neuronal gain, and the underlying mechanisms, are still relatively unknown. The aim of this paper was therefore to fill this knowledge gap by investigating how physiological variables (namely changes in maximal conductance densities) can modulate the gain of neurons, and to elucidate the mechanism by which neurons translate these changes, computationally, to changes in gain.

### The biological significance of gain control from changes in maximal conductances

Our results demonstrate that an increase in the maximal specific conductance (${\bar{G}}_{ion}$) of voltage-gated K^+^ channels including the A-type (*I*_*A*_), delayed-rectifier (*I*_*Kd*_) and Ca^2+^-activated (*I*_*KCa*_) K^+^ channels reduces neuronal gain (Fig. 1), with the *I*_*KCa*_ having the greatest gain-reducing effect (Figs. 1D and 3). In contrast, increases in the slow voltage-gated Ca^2+^ channel conductance (${\bar{G}}_{CaS}$) increases neuronal gain (Fig. 2C), and the hyperpolarization-activated inward channel (*I*_*h*_) is ‘gain-neutral’ (Fig. 2B). Similarly, our theoretical results suggest that changes in passive (non-voltage-gated) conductances lead to parallel shifts in the input-output (current-frequency) relationship, or tuning curve, and do not significantly affect its slope (Fig. 4). This conclusion is consistent with a number of previous studies (e.g. [6, 29–31]), but contrasts with a recent experimental study showing that an increase in passive somatic conductance changes the gain of CA1 pyramidal neurons [11]. There are many possible reasons for this discrepancy; for example, it is possible that the rules of gain modulation in neurons with strong spike-rate adaptation (used in [11]) differ from those without strong spike-rate adaptation (used in our study). The contrasting results could also arise from differences in conductance locations, which can affect firing patterns [33], as well as from synaptic activity, which can change the impact of passive conductances on gain [6, 34].

The findings we present showing that simple changes in the maximal conductances of voltage-gated channels can effect gain control, are potentially of significant biological interest. In biological neurons, conductance densities can change substantially under a variety of physiological and pathophysiological processes, including natural changes in gene transcription (e.g. for the A-type channel, *I*_*A*_[35]), transient brain ischemia (e.g. for the delayed-rectifier K^+^ channel, *I*_*Kd*_[36], and the L-type voltage-gated Ca^2+^ channel [37]), and long-term potentiation (e.g. for the Ca^2+^-activated K^+^ channel, *I*_*KCa*_[38]). In these contexts, the associated changes in gain would enable creation of a spectrum of input sensitivities in a neurochemically uniform population of neurons, thereby increasing the dynamic input range that can be converted to an output. The functional relevance of such changes could include protecting neurons from overexcitation during vulnerable states (e.g. a reduction in gain during ischemia), and for making neurons more excitable following associative learning (e.g. an increase in gain associated with long-term potentiation).

### Biophysical mechanism of gain control in intrinsically-firing neurons

While knowledge of the factors that can lead to gain modulation in neurons is important, in order to understand how changes in neural computation can result in functional changes to neural network activity, we need to first understand the underlying mechanism by which changes in gain are computed. Currently, there is no general mechanism explaining these effects on gain.

Based on our findings, we present the following hypothesis. Gain modulation by changes in ${\bar{G}}_{ion}$ (maximal conductance density) is enabled by changes in the magnitude of current flowing through these channels at particular voltages. This enables changes in the % time the membrane spends at these potentials, thus resulting in a change in the shape of the inter-spike interval (Figs. 8 and 9). For the gain-modulating channels in our model, these effects are partly exerted around the dynamic voltage window (Figs. 8 and 9), which may be a factor of the steepness of channel activation in this voltage range. The combination of these effects means that gain-modulating channels can increase (or reduce) firing rates to a different degree when the drive is strongest. This is summarized by the average current per action potential *vs.* firing rate ($\bar{I}/f$) relationship for these channels (see Fig. 7). For inward currents, a preferential increase in current under depolarizing driving input will manifest as an increase in gain, and a preferential decrease in current under driving input will manifest as a reduction in gain. The opposite will be true for outward currents.

By extension, it should therefore be possible to convert a gain-neutral channel into a gain-modulating channel, for example by increasing the rate of change of that channel’s activation within the dynamic voltage window. To test this, we shifted the steep region of the h-type channel’s (*I*_*h*_, gain-neutral) activation curve into the dynamic voltage window such that activation was half maximal at -50 mV, rather than the baseline -75 mV (Fig. 6B). The shifted activation parameters enabled the channel to modulate gain by exerting a greater depolarizing influence in this voltage window (Fig. 10C, histograms). At higher maximal conductances (${\bar{G}}_{h}$), the depolarizing influence of this channel was increased preferentially at lower firing rates, thus translating to a reduction in gain (Fig. 10B). In theory, such effects could be achieved by changes in either the channel’s steady-state activation variable (as we demonstrate here), the inactivation variable (as we have previously shown for the A-type channel, [15]), or the time constant of activation. Biologically, the former is readily modulated [39–41].

This finding reveals an important new principle of gain control: that tuning the voltage range over which a single channel activates most steeply can influence how effectively the neuron can control input-output ratios, and thus amplify or diminish electrical inputs. This observation provides a new meaning for experimentally-reported shifts in channel activation variables [39–41], in other words, that such shifts can change the computation of how a neuron translates inputs to outputs.

### The biological implications of channel coupling on gain control and the control of excitability

Physiologically, channel coupling is likely to be important in regulating neuronal excitability, as ions flowing through one channel can bind to and inhibit another, and thus be important for neuroprotection. One of the best characterized examples of channel coupling is between [Ca^2+^]-activated K^+^ channels and voltage-gated Ca^2+^ channels, which is the relationship we examined in this paper. Our simulations demonstrate that uncoupling these two channels results in an increased ability of the gain-increaser (*I*_*CaS*_) to increase gain, and a reduced ability of the gain-reducer (*I*_*KCa*_) to reduce gain. This suggests that channel uncoupling results in the neuron being in a more hyper-arousable state. Thus, the physiological function of coupling between these two channels in gain control may be to moderate increases in gain. In addition, we found that increasing the strength of coupling between these channels had a steep gain reducing effect (Fig. 5D). In an analogous way to changes in the ${\bar{G}}_{ion}$ of gain-modulating voltage-gated channels, we found that strong coupling between *I*_*CaS*_ and *I*_*KCa*_ resulted in an increase in the steepness of the *I*_*KCa*_ steady-state activation variable in the dynamic range associated with firing. Thus, the more coupled the two channels, the greater the combined gain-reducing ability, which could be explained by a greater relative contribution of the gain reducer (*I*_*KCa*_). This is consistent with a homeostatic role. Hence, we conclude that although gain control by these two voltage-gated channels is a factor of their intrinsic activation properties, their strength of interaction is also a key factor in gain modulation and the control of excitability. *In vivo*, the effects of voltage-gated Ca^2+^ channels and Ca^2+^-activated K^+^ channels on gain control will likely depend on the distance between the channels, their relative expression levels, amplification of Ca^2+^ entry by Ca^2+^ release from intracellular stores, and the strength of intracellular Ca^2+^ buffering [42–46].

### Implications for different regions of the nervous system

This article focuses on gain control in intrinsically-firing neurons. These correspond to a large class of neurons found throughout mammalian and non-mammalian central nervous systems, including vital neurons of the hypothalamus (e.g. orexin and histamine neurons [47, 48]), midbrain (e.g. dopamine neurons, [35]), raphe nuclei (e.g. serotonin neurons [49]), and thalamus [50]. Cortical neurons, which have been most studied in the context of gain control, have also been reported to fire tonically under certain conditions [51, 52]. We therefore propose that our results on tonically-firing neurons could apply to a variety of important biological neurocircuits.

## Conclusion

In summary, our analysis highlights several previously unexplored principles of gain control in neurons and a new biophysical mechanism by which such gain modulation can be effected. While the ion channel composition and distribution may vary significantly between biological neurons, pathologically or physiologically, the underlying mechanism by which gain control is effected in the neuron model we studied is theoretically generalizable to other neurons with similar spiking properties.

## Supporting Information

S1 Fig The effect of changing membrane capacitance on gain control by voltage-gated channels. Each pair of graphs shows the effect of changing the maximal specific conductance (${\bar{G}}_{ion}$) of a voltage-gated ion channel on gain control, where the specific capacitance of the membrane was fixed at 1 *μ*F·cm^−2^. The left panels shows current-frequency (input-output) relationships obtained with different values of ${\bar{G}}_{ion}$ in the model neuron (maximal specific conductance densities, in mS·cm^−2^, are given near the corresponding tuning curves) when stimulated with different tonic driving currents. Inward, depolarizing, driving current inputs are represented as positive values. The right panels show data in the left panels re-plotted as maximal gain (see Methods) against ${\bar{G}}_{ion}$ where the membrane capacitance was fixed at 1 *μ*F·cm^−2^ (solid lines). Dashed lines represent the same analysis conducted with a membrane capacitance of 0.6 *μ*F·cm^−2^, as in Figs. 1 and 2. This analysis is shown for: (A) the A-type channel, (B) the delayed-rectifier K^+^ channel, (C) the Ca^2+^-activated K^+^ channel, (D) the h-type channel, and (E) the slow voltage-gated Ca^2+^ channel.
(TIF)
