# Supplementary figures and images for: Mechanisms of Gain Control by Voltage-Gated Channels in Intrinsically-Firing Neurons
Source: PLoS One. 2015 Mar 27;10(3):e0115431. doi: 10.1371/journal.pone.0115431 (PMC4376733; doi:10.1371/journal.pone.0115431)

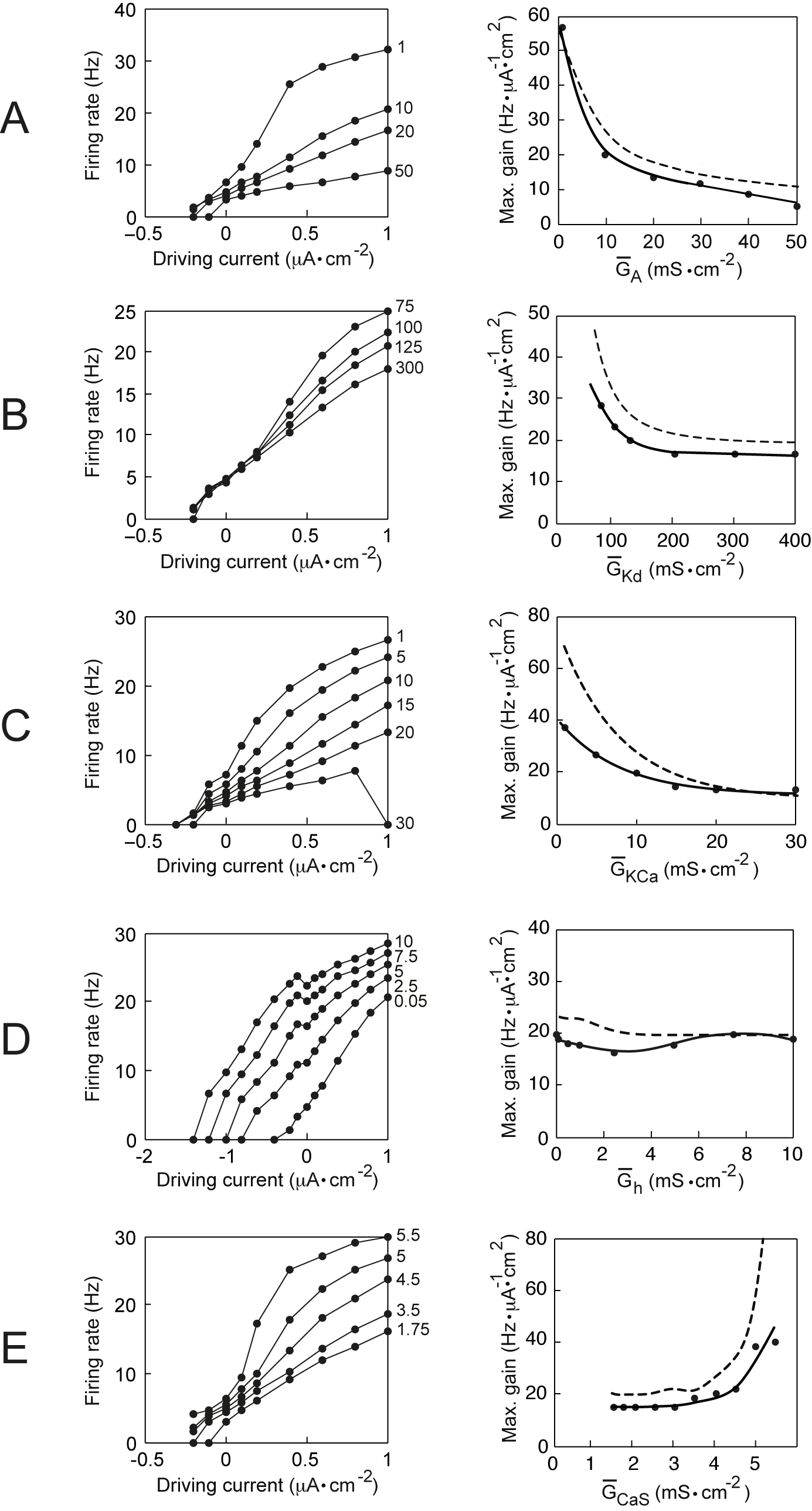

Supplement: S1 Fig — Each pair of graphs shows the effect of changing the maximal specific conductance (G‾ion) of a voltage-gated ion channel on gain control, where the specific capacitance of the membrane was fixed at 1 μF·cm−2. The left panels shows current-frequency (input-output) relationships obtained with different values of G‾ion in the model neuron (maximal specific conductance densities, in mS·cm−2, are given near the corresponding tuning curves) when stimulated with different tonic driving currents. Inward, depolarizing, driving current inputs are represented as positive values. The right panels show data in the left panels re-plotted as maximal gain (see Methods) against G‾ion where the membrane capacitance was fixed at 1 μF·cm−2 (solid lines). Dashed lines represent the same analysis conducted with a membrane capacitance of 0.6 μF·cm−2, as in Figs. 1 and 2. This analysis is shown for: (A) the A-type channel, (B) the delayed-rectifier K+ channel, (C) the Ca2+-activated K+ channel, (D) the h-type channel, and (E) the slow voltage-gated Ca2+ channel. (TIF) [file pone.0115431.s001.tif]
